# Exploring the Link Between Vitamin B Levels and Metabolic Syndrome Risk: Insights from a Case-Control Study in Kazakhstan

**DOI:** 10.3390/jcm13237206

**Published:** 2024-11-27

**Authors:** Alma Nurtazina, Ivan Voitsekhovskiy, Maxat Toishimanov, Daulet Dautov, Kairat Karibayev, Yerbol Smail, Saule Rakhyzhanova, Saltanat Adilgozhina, Bakyt Kanapiyanov, Nurgul Myrzabayeva, Magripa Bapayeva, Altay Dyussupov

**Affiliations:** 1 Department of Epidemiology and Biostatistics, Semey Medical University, Semey 071400, Kazakhstan; alma.nurtazina@smu.edu.kz; 2 Outpatient Clinic #1, Department of Internal Medicine and Cardiology, Semey 071400, Kazakhstan; 3 Faculty of Biology and Biotechnology, Al-Farabi Kazakh National University, Almaty 050040, Kazakhstan; 4 Food and Environment Safety Laboratory, Kazakhstan-Japan Innovative Center, Kazakh National Agrarian Research University, Almaty 050010, Kazakhstan; maxat.toishimanov@gmail.com (M.T.); nurgul.myrzabayeva@kaznaru.edu.kz (N.M.); 5 Department of Propaedeutics of Internal Diseases, Asfendiyarov Kazakh National Medical University, Almaty 050000, Kazakhstan; dautov.d@kaznmu.kz; 6 Central Clinical Hospital, Almaty 050067, Kazakhstan; karibayev@ckb.kz; 7 Department of Infectious Diseases, Dermatology and Immunology, Semey Medical University, Semey 071400, Kazakhstan; erbol.smail@smu.edu.kz; 8 Department of Physiological Disciplines, Semey Medical University, Semey 071400, Kazakhstan; saule.rakhyzhanova@smu.edu.kz; 9 Department of General Practice, Semey Medical University, Semey 071400, Kazakhstan; saltanat.adilgozhina@smu.edu.kz; 10 Department of Propaedeutics of Internal Diseases, Semey Medical University, Semey 071409, Kazakhstan; bakyt.kanapiyanov@smu.edu.kz; 11 1st Clinical Hospital, Almaty, Kazakhstan; info@pervaya.kz; 12 Rector Office, Semey Medical University, Semey 071400, Kazakhstan; altaidusupov@gmail.com

**Keywords:** metabolic syndrome, serum levels of vitamins B, Kazakh population

## Abstract

**Background/Objectives**: Metabolic syndrome (MS) is a collection of metabolic disorders that include insulin resistance, central obesity, dyslipidemia, and hypertension. The prevalence of MS affects 20–30% of adults worldwide, leading to serious health, social, and economic issues. Mitochondrial dysfunction, characterized by mitochondrial DNA (mtDNA) mutations and altered dynamics, plays a pivotal role in MS by impairing glucose oxidation. B vitamins are crucial for optimal mitochondrial function and overall metabolic processes, particularly within the context of MS. This study aims to investigate the associations between plasma concentrations of B vitamins and the risk of MS within the Kazakh population. **Methods**: In this case-control study, biochemistry measurements included serum fasting glucose, HbA1c, creatinine, and lipid profile parameters. The sample comprised individuals who agreed to participate in the investigation and at the Semey polyclinic between December 2022 to March 2024. A total of 190 Kazakhs aged 35–65 years old, including 104 subjects with MS and 86 without MS, took part in the study. **Results**: In a comparative analysis of serum vitamin B levels against established reference ranges, the following results were observed: 95% of participants exhibited vitamin B2 levels at the lower limit of normal, while 4.59% were classified as low. For vitamin B3, 95.77% showed low levels, with only 4.23% in the normal range. Vitamin B6 levels were low in 76.02% of participants. In contrast, 92.82% had normal serum levels of vitamin B9. Regarding vitamin B12, 38.82% had normal levels, 59.41% had elevated levels, and 1.76% were classified as low. Among the evaluated vitamins, only vitamin B2 showed a significant correlation with the risk of developing MS, with an OR of 1.79 (95% CI 1.003, 3.19, *p* = 0.05). **Conclusions**: Relatively elevated serum levels of vitamin B2 at the lower limit of the normal range are associated with a 1.8-fold increased risk of developing MS.

## 1. Introduction

Metabolic syndrome (MS) has emerged as a critical global health concern, with a prevalence of about 25% in the adult population worldwide [1]. The components of MS like obesity, dyslipidemia, hypertension (HT), and insulin resistance independently contribute to cardiovascular risk (CVR) [2,3].

The etiology of MS is multifactorial, encompassing a range of biological mechanisms such as insulin resistance, adipose tissue dysfunction, systemic inflammation, oxidative stress, circadian rhythm disturbances, and changes in gut microbiota, genetic predispositions, and maternal programming [4]. Each of these factors contributes to the pathogenesis of MS, influencing cellular metabolism and energy homeostasis through perturbations in metabolic pathways, including glycolysis and fatty acid oxidation [5,6]. Mitochondrial dysfunction, characterized by mitochondrial DNA (mtDNA) mutations and altered dynamics, plays a pivotal role in MS by impairing glucose oxidation [7,8]. Specific mtDNA-encoded proteins have been linked to prevalent conditions such as type 2 diabetes mellitus (T2DM) and HT, emphasizing the interplay between mitochondrial health and metabolic disorders [9,10].

Aging further complicates the landscape of MS, with prevalence rates exceeding 40% in adults over the age of 60 [11,12]. The aging process is associated with a decline in mitochondrial function, marked by decreased oxidative capacity, increased generation of reactive oxygen species (ROS), and impaired adenosine triphosphate (ATP) synthesis [13,14]. These alterations contribute to a compromised ability to oxidize glucose and fatty acids, thereby undermining insulin sensitivity and metabolic homeostasis [15].

Additionally, B vitamins are crucial for optimal mitochondrial function and overall metabolic processes, particularly within the context of MS [16]. Five out of the eight B vitamins, B1 (thiamine), B2 (riboflavin), B3 (niacin), B5 (pantothenic acid), and B7 (biotin),—are directly involved in the tricarboxylic acid (TCA) cycle, also known as the Krebs cycle [17]. The TCA cycle represents a central hub for energy production, generating nicotinamide adenine dinucleotide (NADH) and flavin adenine dinucleotide (FADH_2_), which facilitate ATP production via oxidative phosphorylation in the electron transport chain [18,19].

The interplay between B vitamins and the risk of MS presents a promising area of study that may enhance our understanding of the syndrome’s pathogenesis and identify modifiable risk factors. Higher vitamin B12 levels correlate with a decreased MS risk, evidenced by a meta-analysis showing a high odds ratio [20,21]. Niacin, as a substrate for nicotinamide phosphoribosyl transferase (NAMPT), plays a crucial role in NAD+ biosynthesis, which is vital for energy metabolism and inflammation regulation [22]. Vitamin B3 influences cellular processes, including insulin signalling and lipid metabolism, which are critical in managing MS [23]. A study utilizing NHANES data found that higher serum levels of vitamin B9 were linked to a lower risk of MS, with odds ratios indicating significant protective effects in higher quartiles of vitamin B9 intake [24]. Higher intakes of vitamin B1, B2, niacin, and vitamin B6 were linked to lower odds of MS, with odds ratios indicating significant protective effects for the highest quartiles of intake [25].

The current literature predominantly focuses on the therapeutic effects of B vitamin supplementation in mitigating the risk of MS [25,26,27,28,29]; however, there is a notable scarcity of research addressing the blood concentrations of B vitamins specifically in the context of MS. Existing studies have primarily focused on isolated components of MS, such as obesity, diabetes, and dyslipidemia, rather than holistic evaluation of MS and blood B2 vitamins levels [30,31,32]. Thus, this study aims to explore the plasma levels of B vitamins and the risk of MS in Kazakh population.

## 2. Materials and Methods

This is a case-control study, conducted in five primary care centers (PHCs) in Semey, in the Abai Region, located in eastern Kazakhstan. Participants were recruited from December 2022 to March 2024. Individuals who met the established inclusion criteria were invited to undergo interviews, physical examinations, and laboratory blood tests. Prior to enrollment, participants received a brief overview of the study objectives and procedures. Enrollment was completed upon the signing of informed consent by each participant.

### 2.1. Sampling

A two-stage sampling design was employed for participant selection. Initially, five out of the forty general practices (GPs) in the city of Semey were randomly selected. Subsequently, sample frames were constructed for potential participants from each selected GP, using lists of patients who met the established inclusion and exclusion criteria. In the second stage of sampling, 50 participants were randomly drawn from each unit. Randomization was conducted using simple random sampling through a computer program designed for generating random numbers.

### 2.2. Participants

Initially, a total of 253 candidates were enrolled in the study, based on a simple random sampling. During the laboratory phase, 63 samples were excluded due to storage issues. Consequently, data from 190 participants, consisting of 104 subjects with MS and 86 subjects without MS, were included in the final analysis.

Inclusion criteria: subjects aged between 35 and 65 years who identified as Kazakh, with both parents of Kazakh ethnicity. Participants were categorized into two groups: those diagnosed with MS and those without the condition (controls). Eligible individuals were required to have refrained from the use of B vitamin supplementation for a duration of twelve months preceding the study. This ensured a homogeneous baseline regarding B vitamin intake among participants, thereby minimizing potential confounding factors related to supplementation. Exclusion criteria included the following: ethnicity other than Kazakh; history of stroke or myocardial infarction (MI), ischemic heart disease, heart failure, thyrotoxicosis, hypothyroidism, diabetes mellitis, or bowel diseases; and the use of statin therapy less than six months prior to the initiation of the study.

### 2.3. Data Collection and Measurements

Standardized questionnaires were employed to gather demographic information, including smoking status, family history, and hereditary predispositions to cardiovascular diseases (CVD) and HT. Measurements of weight, height, and waist circumference were performed in accordance with WHO recommendations [33]. A standardized stadiometer was used for height assessment, while a calibrated scale was utilized for weight measurement on an empty stomach after urination and defecation, without shoes and outwear. BP was measured utilizing the Korotkov method, in accordance with the ESH/ESC algorithm, with participants resting in a seated position during the assessment [34]. Two consecutive measurements were taken for each participant, and the average of these values was recorded. Information regarding history of comorbidities and any medications taken was obtained from medical records as well as through participant interviews. All collected information for each participant was compiled into an individualized file with coded personal data to ensure confidentiality.

### 2.4. Anthropometric Data and Blood Pressure Measurements

BMI was determined using the formula weight in kilograms divided by height in meters squared (kg/m^2^). WC was assessed without applying pressure to the body surface, utilizing a prominent anatomical point on the abdomen for accuracy. To minimize inter-observer variability and enhance measurement reliability, all anthropometric assessments were conducted by a single trained investigator.

### 2.5. Diagnostic Criteria

MS was diagnosed in accordance with the criteria established by the International Diabetes Federation (IDF), which mandates the presence of abdominal obesity along with at least two of the following four clinical factors: systolic blood pressure exceeding 130 mmHg or diastolic blood pressure exceeding 85 mmHg, TG greater than 1.7 mmol/L, HDL-C levels below 1.03 mmol/L in men and below 1.29 mmol/L in women, and plasma glucose concentrations exceeding 5.6 mmol/L.

Obesity was categorized according to the World Health Organization (WHO) criteria based on BMI, which defined the following classifications: normal weight as a BMI of less than 25 kg/m^2^, overweight as a BMI ranging from 25 to 29.9 kg/m^2^, and obesity as a BMI greater than 30 kg/m^2^. Abdominal obesity cutoff points were defined as 94 cm for male subjects and 80 cm for female subjects.

Hypertension was diagnosed following the European Society of Cardiology (ESC) guidelines, ensuring that secondary or symptomatic hypertension was excluded. All participants were documented to have a confirmed diagnosis of essential hypertension in their medical records, were actively taking antihypertensive medications, and were under regular follow-up by their general practitioners.

Dysglycemia, which includes conditions such as impaired fasting glucose (IFG), impaired glucose tolerance (IGT), prediabetes, and diabetes, was defined according to the current recommendations of the American Diabetes Association (ADA). Glycemic status was classified based on hemoglobin A1c (HbA1c) levels, with diabetes indicated by values of 6.5% or higher and prediabetes defined as HbA1c values ranging from 5.7% to 6.4%.

### 2.6. Variables

The main outcome of interest was MS. The primary exposures were vitamins B, including B2, B3, B6, B9, and B12. Factors, such as gender, age, education, history of cardiovascular diseases and hypertension/antihypertensive therapy, smoking, creatinine, and lipid profile parameters like low-density lipoprotein cholesterol (LDL-C) and pulse were considered as potential confounding factors. Serum glucose, hemoglobin A1c (HbA1c), high-density lipoprotein cholesterol (HDL-C), plasma triglycerides (TG), total cholesterol (TC), blood pressure (BP), body mass index (BMI), and waist circumstance (WC) were used for analysis of interrelations with vitamins B.

### 2.7. Ethics Considerations

The research protocol was approved by the Ethics Committee of Semey Medical University on 16 March 2022 (minutes #7). Throughout the study, confidentiality and privacy were strictly maintained. All personal data were anonymized and stored in a secure database accessible only to the project manager and two designated research team members.

Before participating, individuals received detailed information about the study’s aims and objectives. They were informed that participation was voluntary and that they could withdraw at any time without giving a reason and without facing any penalties. Informed consent was obtained from those who met the inclusion criteria.

### 2.8. Biochemistry

Blood samples were collected in the morning following a minimum fasting period of eight to twelve hours, employing intravenous venesection techniques in a standardized manner at the same laboratory by trained nursing personnel affiliated with these facilities for both biochemistry and mass spectrometry analyses. This approach ensured consistency in sample handling and processing across all participants. The laboratory analysis encompassed the quantification of several biochemical markers, including TC, LDL-C, HDL-C, TG, fasting glucose levels, HbA1c, and creatinine concentrations. The assessment of vitamin B levels was conducted using high-performance liquid chromatography coupled with mass spectrometry (HPLC-MS). The specific vitamins analyzed included riboflavin (vitamin B2), niacin (vitamin B3), pyridoxine (vitamin B6), folate (vitamin B9), and cobalamin (vitamin B12).

### 2.9. High Performance Liquid Chromatography Mass Spectrometry 

High-performance liquid chromatography (HPLC) grade methanol and acetonitrile were purchased from Sigma-Aldrich (St. Louis, MO, USA). Acetone was purchased from #1 Chemreactive (Moscow region, Russia). Vitamin standards were commercially obtained from Dr. Ehrenstorfer LGC Standards (Augsburg, Germany). Formic acid was from Sigma-Aldrich.

#### 2.9.1. Standard Solutions

Stock solutions of 1 mg/mL B1, B3, B5 in deionized water, and B2 and B9 in K_2_CO_3_ were prepared in glass containers and stored in +4 °C. Four water-soluble vitamin calibration levels in the range of 1–50 ng/mL were prepared in 0.1% FA in deionized water.

#### 2.9.2. Sample Preparation

Each 100 μL blood serum sample was mixed with 400 μL of methanol:acetone:acetonitrile mixture (1:1:1, *v*/*v*/*v*). The mixture was incubated at 4 °C for 10 min to precipitate proteins. Next, the mixture was vortex-mixed for 30 s and centrifuged for 10 min (15,000 rpm) at 4 °C. The 300 μL supernatant was transferred into a 2 mL eppendorf centrifugation tube and evaporated to dryness by N_2_ gas with sample concentrator (NDK200-1N, Miulab, Hangzhou, China). The dried residue was added to 100 mL of 0.1% FA in deionized water. The final 20 μL solution was taken for HPLC-MS analysis.

#### 2.9.3. HPLC-MS Analysis

The serum vitamin analysis was conducted on a Dionex Ultimate 3000 UHPLC with TSQ Quantum Access Max triple quadrupole mass spectrometer (Thermo Scientific, Austin, TX, USA) and equipped with a degasser SRD-3600, rapid separation binary pump HPG-3400RS, rapid separation thermostatted autosampler WPS-3000TRS, and a rapid separation thermostatted column compartment TCC-3000RS. The serum vitamins were analyzed using reverse-phased C18 silica 150 mm × 4.6 mm × 5 µm and 175 A pore sizes Hypersil GOLD aQ column (Thermo Fisher Scientific, Waltham, MA, USA), which connected with a 4 mm × 10 mm × 5 µm Hypersil GOLD aQ guard column (Thermo Fisher Scientific).

Mobile phases A and B for determination vitamins consisted of deionized water with 0.1% FA and ACN with 0.1% FA, respectively. The flow gradient consisted of the following parameters: 0 min—100% A; 12 min—70% A; 14 min—0% A; 16 min—100% A. The flow rate was 0.6 mL/min. The column oven temperature was 25 °C. The autosampler temperature was set at 10 °C. The triple quadrupole system was performed in positive electrospray ionization mode (ESI). The electrospray voltage was set at 4 kV, the ion source gas 1 (a desolvation gas consisting of nitrogen 99.9%) pressure was set at 20 psi, the ion source gas 2 (a nebulizer gas consisting of nitrogen) was set at 45 psi, and the drying gas (N_2_) flow was 8 L/min. Instrument control, data acquisition, and data processing were performed by XCalibur software, version 4.0 (Thermo Scientific, Austin, TX, USA).

### 2.10. Biases

To minimize selection bias, a two-level random sampling approach was implemented, ensuring that the study group was representative of the target population. Cases and controls were drawn from the same source. To reduce measurement error, blood pressure, height, weight, and waist circumference were measured in the same way using standardized methods by trained staff. Laboratory tests were performed in a single laboratory. Interviews were conducted in a standardized manner by specially trained staff to mitigate observer bias. MS, HT, and obesity were clearly defined and diagnosed according to established criteria prior to the study. The final database was independently double-checked by two members of the research team to prevent systematic input bias.

### 2.11. Sample Size

The sample size was determined to be 170 participants utilizing the Epi-Info 7.1 statistical software. This calculation was based on a confidence interval (CI) of 95%, a statistical power of 80%, and an assumed proportion of exposure among the control group of 25%. The odds ratio (OR) was hypothesized to be 2.5, with a case-to-control ratio of 1:1.

Given the limited and sparse information regarding the proportions of vitamin B levels in individuals with and without MS, an additional 20 participants were incorporated into the sample to enhance the study’s validity. Thus, the total sample size was adjusted to 190 participants to ensure adequate statistical power and reliability of the results.

### 2.12. Quantitative Variables

The status of MS, HT, gender, family history of CVDs, and antihypertensive therapy were classified as binary variables. Smoking status was categorized into three groups: nonsmokers (never smoked), former smokers (who smoked in the past), and current smokers (who are smokers at the moment of interview). BMI was classified as a ranked variable with three categories: normal weight, overweight, and obese. Age was stratified into four groups: individuals younger than 39 years, those aged 40–49 years, those aged 50–59 years, and individuals older than 60 years.

Lipid profile parameters—including TC, LDL-C, HDL-C, TG—as well as vitamin B2, B3, B6, B9, and B12, were analyzed both as binary variables based on the 50th percentile cut-off and as continuous variables.

### 2.13. Statistical Methods

Statistical analysis was performed using STATA Statistical Software, release, version 15, College Station, TX, USA; StataCorp LLC. Continuous variables were reported as means with standard deviation (SD) if they followed a normal distribution. For continuous variables with a highly skewed distribution to the right, a log transformation was applied, provided that the proportion of zero values was less than 2%; otherwise, the median with interquartile range (IQR) was used. Categorical variables were presented as proportions expressed in percentages. For the analysis, chi-squared tests and ORs, along with Mantel-Haenszel ORs with 95% CI, were calculated to examine the associations between categorical risk factors and the primary outcome of interest, comparing MS group with non-MS group. Student’s *t*-test or Welch’s *t*-test was used depending on the normality status of the continuous variables. Differences in population characteristics between comparison groups were assessed using two-sample *t*-tests, signed-rank tests, or chi-squared tests for continuous and categorical variables, respectively. Furthermore, statistical analyses were conducted separately for female and male participants within the datasets. This approach allowed for a detailed examination of potential gender-specific effects and variations in the data.

## 3. Results

The general characteristics of the obtained data are summarized in Table 1. A comparative analysis revealed that subjects diagnosed with MS were older than those in the non-MS group. Furthermore, individuals with MS showed significantly poorer obesity parameters, including BMI and WC, with pronounced effects observed in the female cohort. Specifically, 7.69% of individuals with MS were classified as having a normal weight, while 34.62% were identified as overweight and 57.69% as obese. In contrast, among individuals without MS, a significantly higher proportion (45.35%) were categorized as normal weight, 41.86% = as overweight, and only 12.79% as obese (*p* = 0.0001). In total, 79.01% males and 94.71% females had abdominal obesity. Among individuals diagnosed with MS, 52.11% of males were identified as hypertensive (*p* = 0.0001). In females, the prevalence of HT was markedly higher among those with MS at 80.95%, compared to only 13.95% of females without MS (*p* = 0.0001).

Additionally, participants with MS demonstrated elevated BP levels and a higher prevalence of HT, with a greater proportion of these individuals receiving antihypertensive therapy. Notably, no significant differences were observed between the MS and non-MS groups concerning heart rate and smoking habits.

Table 2 presents a comparative analysis of biochemical data between male and female participants with MS and those without MS. The results indicate that fasting glucose, HbA1c, and TG serum levels were significantly elevated in both males and females with MS compared to their non-MS counterparts. In males, 19.57% of those diagnosed with MS exhibited elevated plasma creatinine levels, compared to 11.76% of males without MS (*p* = 0.350). In females, 9.62% of those with MS presented with elevated plasma creatinine levels, whereas 4.65% of females without MS showed similar elevations. (*p* = 0.192).

A total of 90.2% of participants with MS had hyperglycaemia compared with 47.06% of participants without MS (*p* = 0.0001). The distribution of hyperglycaemia was comparable between females and males within the studied population. Specifically, 30.91% of females exhibited normal glycemia, in contrast to 25.93% of males. Conversely, the prevalence of hyperglycaemia was observed to be 69.09% among females and 74.07% among males. In males, 50.00% with MS and 76.47% without MS had increased plasma levels of LDL-C (above 3.3 mmol/L) (*p* = 0.016). In females, 60.58% with MS and 65.12% without MS, had increased plasma levels of LDL-C (*p* = 0.520). In male, 54.35% with MS and 11.76% without MS, had increased plasma levels of TG (*p* = 0.0001). In females, 44.23% with MS and 8.14% without MS, had increased plasma levels of TG (*p* = 0.0001).

Conversely, HDL-C levels were found to be significantly lower in individuals with MS. However, no significant differences were observed between the groups for other parameters, including creatinine, TC and LDL-C.

### 3.1. Gender-Specific Characteristics of Vitamins B in Relation to MS

Initially, we conducted a comparison of the medians for male and female subjects, independently on MS status, while taking into account the inequality of variances and the distribution characteristics. The results of the Welch’s *t*-test, applied to log-transformed means, indicated a significant elevation in serum levels of vitamins B2, B6, and B9 in males compared to females. However, no differences were observed in vitamins B3 and B12 between the two gender groups (Table 3).

Comparing serum levels of vitamins B with normal reference ranges, 95% of participants exhibited serum vitamin B2 levels at the low limit of the normal range, while 4.59% were classified as having low levels. For vitamin B3, 95.77% of participants presented with low serum levels, with only 4.23% demonstrating normal levels. Regarding vitamin B6, 76.02% of participants had low serum levels, while 23.98% maintained normal levels. Conversely, 92.82% of participants exhibited normal serum levels of vitamin B9, with only 2.56% falling below the normal range (4–35 ng/mL). Concerning vitamin B12, 38.82% of participants had normal levels, 59.41% had elevated levels, and 1.76% was found to have low levels of vitamin B12.

We performed a separate analysis of vitamins B levels in male and female participants, as presented in Table 4 and Table 5. Our findings indicate that serum concentrations of vitamins B3 and B6 were generally observed to be below the normal reference ranges. Conversely, the serum levels of vitamin B9 were normal, while those of vitamin B12 were found to be elevated in relation to these normal ranges. Males exhibited higher overall levels of B vitamins compared to their female counterparts. In male participants, the median levels of vitamins B2 and B12 were found to be higher in those with MS compared to individuals without MS. In contrast, the levels of other vitamins, such as B3, B6, and B9, were similar between the comparison groups. In female participants, levels of vitamins B2, B6, and B12 were also higher in individuals with MS compared to those without the condition. Furthermore, among the female subjects, MS was associated with increased levels of B vitamins, whereas in males, it was associated with decreased levels of these vitamins.

### 3.2. Vitamins B and Risk of Hyperglycemia

We investigated the relationship between various B vitamins and the risk of prediabetes and diabetes. Our analysis indicated that none of the B vitamins demonstrated a significant association with abnormal HbA1c levels. Consequently, we proceeded to adjust the ORs for potential confounding variables, including gender, age, lipid profile parameters, and BMI. Our findings revealed a significant association involving vitamin B9. After adjusting for LDL-C, we observed that participants with an LDL-C level below 3.3 mmol/L exhibited a 2.28-fold increase in the odds of hyperglycemia when their serum vitamin B9 level was equal to or greater than 16.25 ng/mL, as compared to those with lower vitamin B9 levels (OR = 2.28; 95% CI 0.967–5.393; *p* = 0.052).

Similarly, we assessed the role of vitamin B12 in the context of hyperglycemia. After adjusting for HDL-C, individuals with low serum HDL-C levels displayed a 2.85-fold increase in the odds of developing hyperglycemia if their serum vitamin B12 concentration was at least 1.21 ng/mL (OR = 2.85; 95% CI 1.14–7.10; *p* = 0.018).

### 3.3. Vitamins B and Parameters of Lipid Profile

A total of 44.85% of participants, (males and females equally) had LDL-C > 3.3 mmol/L. In total, 35.57% of participants had HDL-C less than 1.3 mmol/L in males (28.57%) and less than 1.3 in females (40.91%), While 28.35% of participants had TG serum level 1.7 and above mmol/L. Levels of TG were increased in 21.82% of females and 36.90% of males (*p* = 0.021).

Investigating lipid profiles among participants, it was observed that 44.85% revealed LDL-C levels exceeding 3.3 mmol/L, with equal representation of both male and female participants. Furthermore, 35.57% of the cohort had HDL-C levels below 1.3 mmol/L; this subgroup comprised 28.57% of males and 40.91% of females. Additionally, 28.35% of participants presented with TG serum levels at or above 1.7 mmol/L. Notably, analysis revealed that 21.82% of females and 36.90% of males demonstrated elevated TG levels, with a statistically significant difference (*p* = 0.021).

We tested all targeted vitamins B for the association with LDL-C, HDL-C, and TGs. Our findings revealed a significant association solely between vitamin B2 and triglyceride serum concentrations. An increase in vitamin B2 beyond 2.15 ng/mL was associated with 1.67 times greater odds of developing hypertriglyceridemia when compared to individuals exhibiting lower serum levels of vitamin B2 (OR = 1.84; 95% CI = 0.96;3.52; *p* = 0.06).

Furthermore, after adjusting for hyperglycemia, the association between vitamin B2 and hypertriglyceridemia showed a significant enhancement (OR = 1.91; 95% CI = 0.98;3.75; *p* = 0.052).

### 3.4. Vitamins B and Risk of MS and Role of Co-Factors

In this study, we examined the relationship between vitamins B2, B3, B6, B9, and B12 and the risk of MS, as presented in Table 6.

Among the vitamins assessed, only vitamin B2 exhibited a significant association with the risk of developing MS, yielding OR of 1.79 (95% CI 1.003, 3.19, *p* = 0.05). Upon adjusting for LDL-C levels, this association appeared to strengthen slightly, resulting in an OR of 1.82 (95% CI 1.01, 3.29, *p* = 0.042). To specify, within the cohort with LDL-C serum levels less than 3.3 mmol/L, the association between elevated serum levels of vitamin B2 and the risk of MS was found to be 2.27 times stronger in comparison to individuals with serum levels of vitamin B2 below 2.15 ng/mL (95% CI 1.01, 5.12, *p* = 0.042). Conversely, in the group with LDL-C levels equal to or exceeding 3.3 mmol/L, no significant association was observed between elevated vitamin B2 levels and MS (95% CI 0.59, 3.36; *p* = 0.44).

An initial crude analysis did not reveal a significant association between vitamin B3 and MS. However, following adjustment for LDL-C, a notable enhancement in the association emerged. Specifically, when serum levels of vitamin B3 reached or exceeded 0.28 ng/mL, and after accounting for potential confounding effects of LDL-C, the odds of developing MS nearly doubled, as indicated by an OR of 1.82 (95% CI1.01, 3.29, *p* = 0.043).

Additionally, the crude association between serum levels of vitamin B12 and the risk of MS was not statistically significant (OR = 1.80, 95% CI 0.95, 3.41, *p* = 0.06). However, following adjustment for low HDL-C, this association achieved statistical significance, with an OR of 2.0 (95% CI 0.97, 3.94, *p* = 0.05).

We investigated the association between serum concentrations of vitamin B2 and the risk of MS, considering the influence of other B vitamins (Table 7). The association between vitamin B2 levels and MS risk was found to be more pronounced when adjusted for vitamin B3, compared to adjustments made for vitamins B6, B9, and B12.

## 4. Discussion

The primary research question addressed in this study refers to the relationship between serum concentrations of B vitamins and the risk of MS. Our results indicate that elevated levels of vitamin B2 are associated with a 1.79 OR of developing MS. This finding stands in contrast to existing literature, which reports an inverse association between vitamin B2 levels and the risk of MS [20,21]. These associations appear to be enhanced by impact of LDL-C and vitamin B3.

This study is the attempt to represent the first comprehensive investigation of the relationships between B vitamins and MS, considering a range of potential co-factors.

The existing literature on the relationship between serum concentrations of B vitamins and MS is relatively sparse, with the majority of studies concentrating on the associations between these vitamins and various components of MS, such as obesity, diabetes, and dyslipidemia. A significant proportion of research has focused on the impact of vitamin B supplementation on MS risk rather than direct associations with serum concentrations [25,27,28,29].

In our study cohort, concentrations of vitamin B2 in both males and females demonstrated that 91.33% of subjects exhibited levels below 6 ng/mL, a threshold considered to be within the low normal range [35] and even below reference values established by some researchers [36,37]. Consequently, based on the results of our investigation, we can conclude that relatively higher instances of low vitamin B2 levels are associated with an increased risk of MS.

Low B2 serum levels despite acting fortification program in Kazakhstan since 2004 can be explained by interference with other possible causes of riboflavin deficiency like high prevalence of thyroid disorders related to endemic iodine deficiency, and undiagnosed steatohepatitis etc.

Vitamin B2 is a precursor of flavin adenine dinucleotide (FAD) and flavin mononucleotide (FMN), which are coenzymes for various dehydrogenase enzymes in the TCA cycle, such as succinate dehydrogenase. Liu et al. identified a correlation between low blood riboflavin levels and an increased risk of T2DM among the Han ethnicity, although the underlying mechanisms remain poorly understood [38]. Riboflavin is essential for ATP production, serving as a precursor for FAD and FMN, which are crucial cofactors for mitochondrial flavoproteins. These flavoproteins are integral to the mitochondrial respiratory chain and β-oxidation enzymes, key components in mitochondrial energy production [39,40]. Moreover, riboflavin functions as a cofactor for various mitochondrial enzymes involved in intermediary metabolism, including amino acid, purine, and fatty acid metabolism [41,42].

In our study, we revealed associations between B2 levels and dyslipidemia like increase of LDL-C and hypertriglyceridemia. We also showed that the process interrelates with glycemic metabolism. These data are consistent with experimental research that demonstrated that deficiency in riboflavin results in decreased FAD levels, adversely affecting fasting glucose availability by impairing the activation of genes regulated by the nuclear receptor PPARα. This dysfunction may lead to hypoglycemia and fatty liver disease, as demonstrated in riboflavin-deficient mice. Additionally, riboflavin deficiency disrupts gluconeogenesis, a critical process for maintaining blood glucose levels during fasting, due to diminished activation of gluconeogenic genes [43].

The altered expression of these key metabolic enzymes promotes the accumulation of triglycerides within the hepatic tissue, thereby facilitating the progression of hepatic steatosis [44].

In riboflavin-deficient rats, both mRNA and protein levels of Apolipoprotein B100 were markedly diminished compared to controls. ApoB100 is essential for lipid transport, constituting significant portions of apolipoproteins in VLDL and LDL. Oxidative folding of ApoB100 in the ER is necessary for its maturation before cytoplasmic secretion. Riboflavin deficiency in rats was induced through specific dietary manipulation. The expressions of ERO1 and PDI, crucial for ApoB100’s oxidative folding, were also significantly reduced at the protein level. Concurrently, serum levels of total cholesterol and triglycerides decreased, while liver concentrations increased in riboflavin-deficient rats [45].

ApoB secretion was reduced in riboflavin-deficient cells relative to other conditions [46].

Riboflavin is absorbed in the small intestine by the human riboflavin transporters RFVT1 and RFVT3. A third riboflavin transporter (RFVT2) is expressed in the brain [47]. Studies have shown that riboflavin deficiency leads to the overexpression of RFVTs in cardiomyocytes, indicating an adaptive response to maintain riboflavin levels [48]. The primary gut microbiota responsible for riboflavin production in humans includes specific strains of *Bifidobacterium* and *Lactobacillus* [49]. Aging is associated with a odecline in these bacteria. In T2DM patients, there is a notable reduction in Lactobacilli spp., linked to insulin resistance [50]. Additionally, a strain of *Bifidobacterium* as a probiotic has been shown to prevent obesity-associated dyslipidemia in mice [51,52].

We found that increased serum concentrations of vitamin B12 are associated with the risk of prediabetes and diabetes when taking into account the potential negative confounding effect of abnormal HDL-C levels. These results are in agreement with the findings reported by He et al. [53]. In contrast, the majority of the existing literature suggests an inverse relationship between serum vitamin B12 levels and the risk of developing diabetes [54].

**Figure 1:**
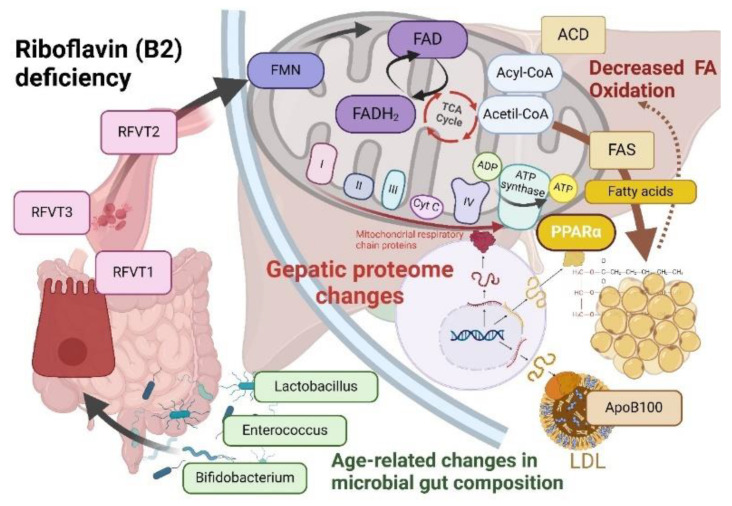
Pathogenetic insights into the association between vitamin B2 and MS. The gut microbiota responsible for riboflavin synthesis in humans includes specific strains of *Bifidobacterium* and *Lactobacillus*. Aging is associated with a decline in these beneficial bacteria, while individuals with type 2 diabetes mellitus (T2DM) show reduced *Lactobacillus* spp., correlating with insulin resistance. A *Bifidobacterium* strain has been shown to reduce obesity-related dyslipidemia in murine models. Moreover, the prevalence of gut Enterobacteriaceae increases with age, independent of genetic or lifestyle factors, contributing to aging through disruption of cellular pathways and mitochondrial dysfunction. Riboflavin absorption occurs in the small intestine via the RFVT1 and RFVT3 transporters, with RFVT2 present in the brain. Riboflavin deficiency induces overexpression of RFVT transporters in cardiomyocytes, indicating an adaptive response for maintaining riboflavin homeostasis. Riboflavin is essential for the synthesis of FAD and FMN, crucial cofactors for mitochondrial flavoproteins involved in the respiratory chain and β-oxidation enzymes necessary for energy production. In riboflavin-deficient rats, Apolipoprotein B100 mRNA and protein levels were significantly reduced compared to controls, highlighting the role of ApoB100 in lipid transport and its association with VLDL and LDL. Additionally, riboflavin deficiency in Pekin ducks markedly affects liver protein profiles, with decreased proteins linked to fatty acid β-oxidation and mitochondrial electron transport, while proteins associated with triacylglycerol and cholesterol synthesis are elevated. Furthermore, mice deficient in vitamin B2 exhibit reduced activation of PPARα target genes, which are critical for gluconeogenesis, suggesting that vitamin B2 deficiency impairs glucose production during fasting.

Our hypothesized mechanism of interrelations between vitamin B2 and MS is presented in Figure 1.

Several limitations of our study should be considered when interpreting and extrapolating the findings to the general population.

1. Inclusion criteria for control group: The inclusion criteria for the control group may have contributed to insufficient precision in delineating the differences between vitamin B levels and MS status. The control group comprised not only apparently healthy subjects but predominantly individuals who did not meet eligibility criteria for MS diagnosis. For instance, both groups include equal proportions of participants with overweight (50%).
2. Reference ranges for vitamin B levels: Our study faced constraints due to the lack of internationally available reference ranges for serum levels of vitamin B, as determined by mass spectrometry, both globally and specifically within Kazakhstan.
3. Absence of dietary assessment: We did not investigate the dietary habits of participants, which could have enhanced the accuracy of our results by accounting for additional potential risk factors.
4. Comorbidity considerations: Information regarding thyroid disease as comorbidity was not collected in women without history of thyroid disorders. However, undiagnosed thyroid disorders may confound the association between riboflavin levels and the risk of MS, given that such conditions can lead to vitamin B2 deficiency. The prevalence of thyroid disorders is notably high in Kazakhstan, an area endemic for iodine deficiency.
5. Lack of data on steatohepatitis: Data on the presence of steatohepatitis in obese participants were not available. This condition is also associated with vitamin B2 deficiency and could potentially interfere with the relationship between B2 levels and MS. Notably, steatohepatitis is reported to be more prevalent in Asian populations compared to their Caucasian counterparts.
6. Dyslipidemia considerations: We did not account for participants’ histories of statin use exceeding six months, which could impact LDL-C levels in the non-MS group.
7. Measurement of obesity: Obesity, a principal component of metabolic syndrome, was quantified solely through BMI and WC based on WHO and IDF criteria for European populations. Kazakhstan’s adherence to European clinical guidelines regarding obesity, applicable across all ethnic groups, may not fully encapsulate the complexities surrounding the relationship between obesity and vitamin B levels, particularly in Asian populations including Kazakhs. Furthermore, it is noteworthy that Kazakhs exhibit anthropometric similarities to Caucasians; however, there is currently a lack of data regarding visceral adiposity specific to this population. Such data could provide valuable insights into which diagnostic criteria for obesity would be most appropriate to apply in this context. The need for research addressing these gaps is imperative to enhance the accuracy and relevance of clinical assessments of obesity and its associated metabolic implications in the Kazakh population.

In summary, these limitations highlight the necessity for cautious interpretation of our results and emphasize the need for further research that addresses these critical areas to elucidate the relationships between vitamin B levels, obesity, and metabolic syndrome more accurately.

## 5. Conclusions

The findings of this study indicate that individuals exhibiting relatively elevated levels of vitamin B2 within the low-normal range are at a significantly increased risk of developing MS, with a 1.8-fold elevation in risk. This association suggests that riboflavin levels, even within the lower end of the normal range, may play a critical role in metabolic health. Further research is warranted to elucidate the mechanisms underlying this relationship and to assess the potential of vitamin B2 as a biomarker for MS development. These results emphasize the importance of monitoring vitamin B2 levels as part of a comprehensive approach to metabolic health and disease prevention.

## Figures and Tables

**Table 1:** General characteristics of the study participants with and without MS.

	MS	Non-MS	*p*-Value
Variables	104	86	
Age (yrs) Mean (SD)	52.72 (7.10)	49.94 (7.72)	0.01
Gender			
Male, *n* (%)	46 (57.50)	34 (42.50)	0.5
Female, *n* (%)	58 (52.73)	52 (47.27)	
BMI (kg/m^2^)			
Male, mean (SD)	27.42 (4.49)	24.52 (4.38)	0.1
Female, mean (SD)	27.63 (6.73)	23.56 (5.59)	0.01
WC (cm)			
Male, mean (SD)	111.44 (13.36)	99.59 (12.15)	0.0002
Female, mean (SD)	102.17 (11.42)	90.24 (9.88)	0.0001
BPsyst (mmHg)			
Male, mean (SD)	131.96 (14.94)	122.29 (13.07)	0.004
Female, mean (SD)	128.27 (16.56)	112.24 ± 17.69	0.0001
BPdiast (mmHg)			
Male, mean (SD)	86.25 (10.19)	80.09 (10.15)	0.01
Female, mean (SD)	79.61 (10.14)	71.61 (11.75)	0.0005
HR (bpm)			
Male, mean (SD)	75.44 (8.60)	74.92 (9.03)	0.8
Female, mean (SD)	73.78 (8.17)	73.39 (9.22)	0.8
Smoking habit			
Yes, *n* (%)	18 (26.47)	39 (33.05)	0.7
No, *n* (%)	50 (73.53)	79 (66.95)	0.3
HT			
Yes, *n* (%)	35 (51.47)	26 (22.04)	0.0001
No, *n* (%)	33 (48.53)	92 (77.96)	0.0001
Antihypertensive therapy			
Yes, *n* (%)	30 (44.12)	16 (13.59)	0.0001
No, *n* (%)	38 (55.88)	102 (86.44)	0.0001

Comparisons between MS and non-MS groups were conducted by using Student’s *t* test for normally distributed continuous data and Fisher’s exact test for categorical data. BMI, body mass index; WC, waist circumference; BPsyst, systolic blood pressure; BPdiast, diastolic blood pressure; HR, heart rate; HT, hypertension; n, number; SD, standard deviation.

**Table 2:** Summary of laboratory data of study participants with and without MS.

Biochemistry Parameters, mmol/L	Males		Females	
	MS, Mean (SD)	Non-MS, Mean (SD)	MS, Mean (SD)	Non-MS, Mean (SD)
Creatinine	96.22 (18.39)	87.83 (17.14)	65.65 (25.75)	63.21 (13.62)
Glucose	4.98 (2.00)	4.52 (1.31)	6.50 (2.99)	4.57 (0.74) *
HbA1c, %	6.27 (0.87)	5.88 (0.57) *	6.73 (1.63)	5.72 (0.45) *
TC	5.39 (1.05)	5.05 (0.76)	5.33 (0.98)	5.08 (0.98)
HDL-C	1.05 (0.28)	1.25 (0.26) *	1.25 (0.25)	1.54 (0.27) *
LDL-C	3.14 (0.87)	3.31 (0.69)	3.34 (0.77)	3.21 (0.84)
TG	3.59 (1.03)	1.39 (0.69) *	1.87 (1.09)	1.04 (0.42) *

Comparisons between metabolic syndrome and non-metabolic syndrome groups were conducted using Student’s *t* test for normally distributed continuous data. * *p*-value of <0.001. HbA1c, glycated hemoglobin; TC, total cholesterol; HDL-C, high-density lipoprotein cholesterol; LDL-C, low-density lipoprotein cholesterol; TG, triglycerides.

**Table 3:** Comparisons of serum levels of vitamins B in males and females *.

Vitamins B, ng/mL	Observations, n	Log-Transformed Mean (SD)	95% CI	*p*-Value
B2				
Females	110	0.75 (0.54)	0.65, 0.85	0.042
Males	80	0.91 (0.52)	0.79, 1.02	
B3				
Females	106	−1.03 (1.09)	−1.24, −0.83	0.122
Males	80	−1.25 (0.78)	−1.42, −1.07	
B6 > 2.16				
Females	110	0.62 (0.60)	0.50, 0.73	0.003
Males	80	0.86 (0.52)	0.75, 0.98	
B9				
Females	109	2.72 (0.57)	2.62, 2.83	0.052
Males	80	2.87 (0.49)	2.77, 2.98	
B12				
Females	91	0.39 (1.31)	0.12, 0.67	0.364
Males	76	0.57 (1.18)	0.30, 0.84	

* Welch’s *t*-test; n, number of observations; SD, standard deviation.

**Table 4:** Characteristics of vitamins B in males with and without MS.

Vitamins B, ng/mL	MS, n (%)			non-MS, n (%)		
	46 (57.5)			34 (42.5)		
	Median (IQR)	Mean (SD)	Range	Median (IQR)	Mean (SD)	Range
B2	2.28 (1.72–2.96)	2.69 (1.75)	0.73–12.05	2.17 (1.83–3.61)	3.22 (2.67)	0.98–12.88
B3	0.26 (0.16–0.40)	0.34 (0.29)	0.61–1.39	0.27 (0.21–0.47)	0.52 (1.06)	0.11–6.92
B6	2.47 (1.71–3.22)	2.56/1.07	0.25–6.25	2.53 (1.94–3.20)	2.64 (1.14)	0.29–5.74
B9	18.25 (13.77–24.12)	19.11/7.59	3.62–43.54	17.62 (12.55–22.68)	19.63 (10.56)	3.05–50.07
B12	1.55 (0.75–3.85)	3.69/5.07	0.33–24.17	1.43 (0.73–4.59)	3.71 (4.70)	0.87–15.82

IQR, interquartile range; SD, standard deviation.

**Table 5:** Characteristics of vitamins B in females with and without MS.

Vitamins B, ng/mL	MS, n (%)			non-MS, n (%)		
	58 (52.7)			52 (47.3)		
	Median (IQR)	Mean (SD)	Range	Median (IQR)	Mean (SD)	Range
B2	2.20 (1.64–2.87)	2.67 (1.86)	0.56–11.42	1.89 (1.45–2.44)	1.99 (0.80)	0.46–4.87
B3	0.28 (0.21–0.52)	0.96/2.10	0.07–9.42	0.25 (0.17–0.44)	0.71/1.45	0.81–7.77
B6	2.05 (1.64–2.88)	2.20/0.95	0.13–5.61	1.86 (1.45–2.27)	1.98/0.85	0.75–4.95
B9	16.13 (11.29–21.56)	17.31/9.23	1.82–63.12	15.00 (11.68–18.60)	15.70/6.29	4.18–38.00
B12	1.25 (0.69–3.43)	5.61/11.95	0.15–37.06	0.85 (0.56–1.49)	1.87/2.97	0.18–15.82

IQR, interquartile range; SD, standard deviation.

**Table 6:** ORs for MS crude and adjusted (Mantel–Haenzel) for increased levels of LDL-C, HDL-C, and TG ^α^.

Vitamins, ng/mL	OR for MS			
	Crude	Adjusted for		
		LDL-C ≥ 3.3 mmol/L	HDL-C ≤ 1.03 mmol/L in Males, ≤ 1.29 mmol/L in Females	TG ≥ 1.7 mmol/L
B2 ≥ 2.15	1.79 *	1.82 *	1.84	1.59
B3 ≥ 0.28	1.18	1.82 *	1.1	1.0
B6 ≥ 2.16	1.38	1.40	1.61	1.22
B9 ≥ 16.25	1.60	1.58	1.53	1.61
B12 ≥ 1.21	1.80	1.76	2.0 *	1.70

^α^ Chi-squared test for categorical variables. * *p* < 0.05. LDL-C, low density lipoprotein cholesterol; HDL-C, high density lipoprotein cholesterol; TG, triglycerids.

**Table 7:** ORs for MS crude and adjusted (Mantel–Haenzel) for increased levels of vitamins B3, B6, B9 and B12 ^α^.

OR for MS				
Crude	Adjusted for: (OR, 95%CI, *p*-Value)			
B2 ≥ 2.15	B3 ≥ 0.28 ng/mL	B6 ≥ 2.16 ng/mL	B9 ≥ 16.25 ng/mL	B12 ≥ 1.21 ng/mL
1.79 *	1.98 *	1.70	1.63	1.81
1.003, 3.19	1.09, 3.61	0.94, 3.08	0.84, 3.17	0.91, 3.63
0.05	0.02	0.07	0.15	0.08

^α^ Chi-squared test for categorical variables. * *p* < 0.05.

## Data Availability

The dataset is available on request from the authors. The raw data supporting the conclusions of this article will be made available by the authors on request.
